# Effectiveness of Glycyrrhizinic Acid (Glizigen) and an Immunostimulant (Viusid) to Treat Anogenital Warts

**DOI:** 10.5402/2012/863692

**Published:** 2012-08-14

**Authors:** José Domínguez Gómez, Ramón Daniel Simón, Alfredo Abreu Daniel, Hana Zelenkova

**Affiliations:** ^1^Hospital Clínico Quirúrgico Comandante Manuel Fajardo, Havana, Cuba; ^2^Private Clic of dermatovenerologie, Svidnik, Slovakia

## Abstract

Genital warts are benign proliferations of skin and mucosa caused by the human papillomavirus infection (hereinafter referred to as HPV). It is one of the most common sexually transmitted diseases in the world, whose incidence rate has increased in the last three decades. Current treatment involves the physical destruction of the infected cells. The fact that there are many different types of treatment goes to show that none of them are uniformly effective or directly antiviral. *Objective*. Demonstrate the efficacy of Glizigen in the III-phase clinical trial combined with a food supplement (VIUSID) formulated to boost the immune system when treating external anogenital warts. *Design*. 100 patients clinically diagnosed with anogenital lesions were included in the trial and assigned to two groups of 50 individuals. Those from one group where treated with Glizigen and Viusid and those from the other group with 25% podophyllin in alcohol, the results from each were then compared. *Results*. The combined Glizigen-Viusid treatment was seen to have an 87.5% efficacy rate, which was slightly more than that of the treatment with podophyllin, and there were hardly any adverse reactions reported during the treatment. *Conclusions*. the combined Glizigen-Viusid treatment was effective in treating genital warts.

## 1. Introduction

Genital warts are benign proliferations of skin and mucosa caused by the human papillomavirus infection. These viruses do not have any acute signs or symptoms, just the slow growth of lesions that can be subclinical for long periods of time [1]. They are a group of DNA viruses that belong to the Papovaviridae family, they are nonenveloped, and they cause all kinds of warts and a great deal of tropism on different parts of the body [2]. Up to 150 different types have been identified and around 40 of these infect the anogenital tract in men and women [3]. They are classified as being either high or low oncogenic risk types, the latter includes types 6 and 11, which are responsible of the appearance of condylomata in more than 90% of the cases [4].

Genital warts is one of the most common sexually transmitted diseases in the world, its incidence rate has increased in the last three decades; 600 million individuals are thought to be infected and 190 million with clinical infection. There are not any official statistics on the prevalence of the HPV infection in Latin America [5]. In Cuba, 83,521 cases were reported in the period 1990–2003 [6].

It is transmitted through sexual intercourse, trauma with contaminated material, contact with surfaces that are infected with the virus, and, occasionally, vertical transmission (trans placental) or perinatal (contact with the birth canal). The incubation period is between 2 to 9 months [7–9]. The disease is not as contagious in the early stages [10–12]. 

Lesions are located on the penis, vulva, scrotum, perineum, and the perianal area; the cervix, urethra, anus, mouth; and also in the conjunctiva, the nose, and the larynx [7]. They vary in appearance; some are pinpoint papules, others are cauliflower-like masses. They can be red, pink, or skin colour [13]. Depending on the size and the anatomical location they can be painful, friable, or itchy. Even though they are usually asymptomatic, they do have a considerable psychological impact on the sufferer.

The diagnosis is based on the clinical examination. The use of acetic acid (3–5%) improves the detection of these lesions, biopsy [7], detecting low serum antibody titres, immunohistochemical analysis of the HPV structural proteins (confirmatory method) by DNA hybridization technique and finding the nucleic acid of a virus (DNA or RNA) or the capsid protein as a definitive diagnosis [11].

Current treatment involves the physical destruction of the infected cells, but the fact that there are many types of treatment goes to show that none of these are uniformly effective or directly antiviral. As the preferred treatment involves eliminating the visible wart, there is no evidence that shows that the existing treatment available eradicates the HPV infection, reduces the infectivity, or modifies the natural history of the infection [11].

The nonsurgical therapy includes the interferon (topical, intralesional, or systematic), podofilox, and imiquimod topical, and the most used (more experience) are podophyllin and the trichloroacetic acid. There are others like intralesional bleomycin, retinoic acid, cantharidin, 5-fluorouracil cream, and the interferon alfa-2b. 1% of Cidofovir, HSPE [14, 15], HPMPC (the analogue of the acyclic nucleoside phosphonates) [12, 16], arotinoid ethyl ester (3rd generation polyaromatic retinoids) [13], zinc sulfate (10 mg/kg/day) [17], dinitrochlorobenzene (DNCB), and the diphenylcyclopropenone (which are thought to stimulate local immunity). Recently, cimetidine has been distinguished for being successful in treating skin warts, especially in children. Cytokines [1] and intralesional injection of Candida antigen by immunotherapy are also administered [18]; not long ago (June 2006) the FDA (Food Drugs Administration) approved the vaccine, that the pharmaceutical company Merck sells under the name of GARDASIL (it blocks the 4 HPV strains of the infection), that can be used on women and girls aged between 9 and 26 [19].

Surgical treatment includes cryotherapy, surgical excision and cryosurgery with laser, the CO_2_ laser, and electrofulguration [1, 3, 4, 7].

The types of HPV causative agents [20], the appearance of the infection, and how it is treated are the same in Cuba as in the rest of the world. The most commonly used specific treatments are: Podophyllin (10–25%) in alcohol solution or in tincture of benzoin, bi- or trichloroacetic acid (80–90%) in alcohol solution, electrofulguration (in isolated lesions), cryotherapy, and surgical exeresis [21–23]. In 2005, a clinical trial was carried out to determine the efficacy of podophyllin, trichloroacetic acid, and cryosurgery although significant differences were not found when these were used to treat external anogenital warts. 

Alternative therapy is part of the medical arsenal available to treat many of the diseases that we are faced with on a daily basis. By using the aforementioned knowledge and that obtained from other recent studies, Catalysis Laboratories has developed a product called GLIZIGEN. One of its main active ingredients is glycyrrhizinic acid, which is a substance found in the *Glycyrrhiza glabra roo*t, commonly known as “sweet root”, that was used way back in ancient Egypt and China to treat respiratory infections and as an anti-inflammatory. Modern-day research has demonstrated its anti-inflammatory activity, its antiulcerative and antiviral effect. The former (0.1 g in 100 mL of the vehicle) interacts with viral proteins, which according to the stage of the viral infection can lead to the inactivation of extracellular free virus particles, the prevention of the intracellular decapsulation of infectious particles, and the deterioration of the assembling capacity of the structural components of the virus.

Its beneficial effects are enhanced by molecular activation that considerably improves the biological activity of the antioxidizing molecules and that of all other molecules that contain carboxyl groups in their structure; it can also make antiviral activity up to 10,000 times as effective.

Clinical studies carried out at dermatovenerology and gynecology clinics confirm its effectiveness. It does not produce any complications in pregnant women; it's 100% effective in the early stages of the disease and 90% in relapse cases; it is very well tolerated, it does not cause skin irritation, and it is quite innocuous. No interactions with other pharmacological preparations have been documented. One of these is Viusid, a food supplement that has been especially designed to help balance and stimulate the functions of the immune system. It contains antiviral agents, antioxidants, and anti-free radicals that are essential for our health as they boost the defenses of the immune system and strengthen the whole organism.

Given the prevalence of this STI, its biological and psychological repercussions, the lack of effective medicine, and the new therapeutic possibilities that provide us with alternative types of treatment, a study was organised so as to evaluate how successful the combination of Glizigen-Viusid is in treating external genital warts.

## 2. Method

A prospective experimental clinical trial was performed with the help of Catalysis Laboratories (Madrid, Spain). The sample consisted of 100 patients who were divided up into 2 groups, each having 50 individuals who were selected according to the inclusion criteria and by simple random sampling.

### 2.1. Method of Use for the Medicine

The lesions of all the patients were measured with a ruler and the area of skin affected was determined. Up to 10 cm^2^ was the limit set for members of both groups.

Members of the control group were administered podophyllin once a week for 6 weeks. At the end of this period, if the lesions had not disappeared, the treatment was changed and the subsequent results were not included in the clinical trial, those obtained during the follow-up period were not either because the patients were considered to have abandoned the clinical trial.

Members of the group that was treated with Glizigen were given written instructions on how many sprays of the product had to be applied at the out-patients unit (once spray is equal to two small squirts) according to the area of skin that was affected: 1 to 3.9 cm^2^, 3 times a day; from 4 to 6.9 cm^2^, 4 times a day; 7 to 10 cm^2^, 5 times a day, for 8 weeks. After this period, the treatment was reviewed to see whether it should be combined or replaced by a more conventional type of treatment that depended on the specific patient, just like the procedure followed with podophyllin, in which case they were considered to have abandoned the clinical trial. Moreover, a 30 mL dose of Viusid (syrup) was administered every 8 hours, each day, during the treatment. The patients were told to take it after meals, dissolving it in drinking water, fruit juice, or milk.

Two patients from the group treated with Glizigen decided, on their own free will, to leave the clinical trial, so 48 patients managed to complete the treatment. All the members from the control group completed the treatment.

The lesions were photodocumented at the start and at the end of the treatment. A data collection model was used (see annex) that showed the general data about the patients and the disease. The results were kept on an Excel database and were statistically processed with the EPIDAT 3.1. software.

## 3. Results

**Figure 1:**
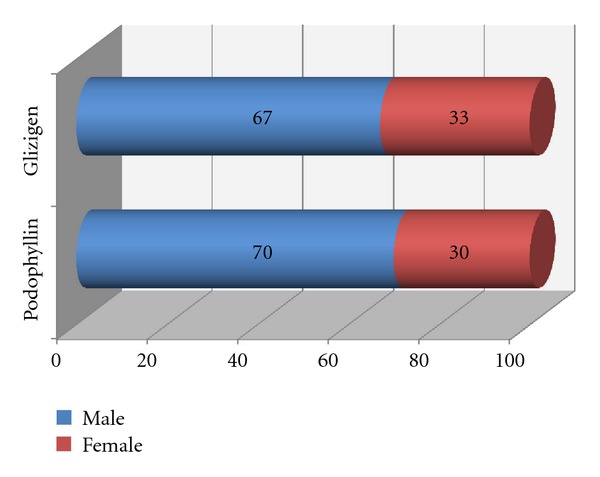
Gender distribution. Hospital Manuel Fajardo, Havana. 2008.

On analysing the distribution of patients according to their age, the predominant group was clearly that consisting of patients aged between 15 and 24 (Table 1). There were no significant differences with regard to the gender-related distribution in either of the products used (Figure 1). 

**Figure 2:**
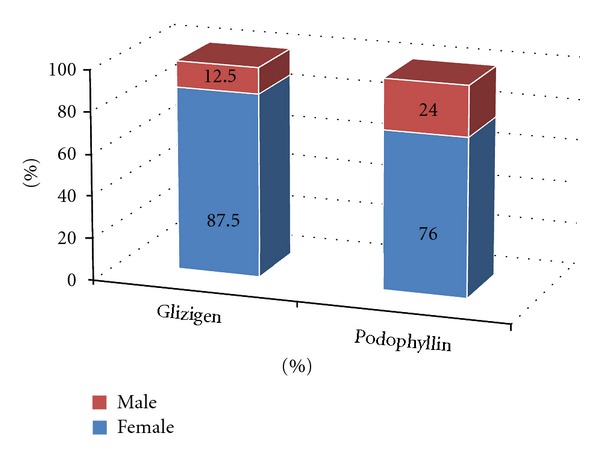
Response to therapy Hospital Manuel Fajardo, Havana. 2008

**Figure 3:**
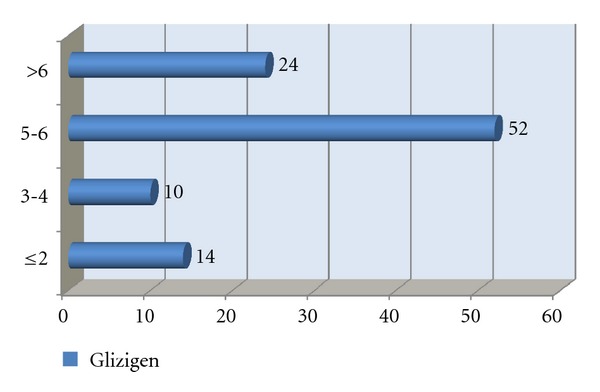
Distribution of patients according to their response to Glizigen in weeks.

**Figure 4:**
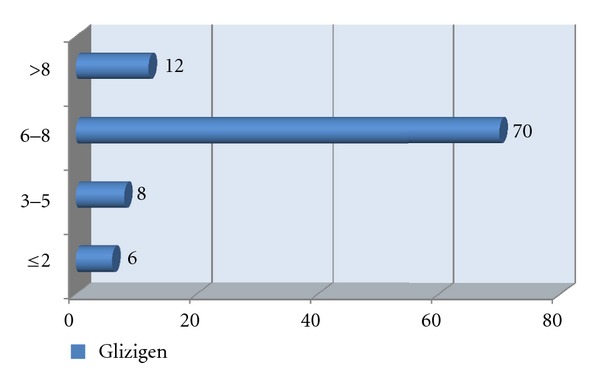
Distribution of patients according to their response to podophyllin in weeks.

Figure 2 shows the response to the treatment; a good response to both products was observed. However, the best response to both treatments was seen in the last two weeks of using them (Figures 3 and 4). 

On evaluating the correlation between curing the skin lesion and the affected area on diagnosis, less than 5 cm^2^ of the skin in more than 84% of the members of both groups was affected (Table 2). With regards to discomfort mentioned by the patients during the treatment period, Glizigen was seen to be tolerated better (Table 3). 

**Figure 5:**
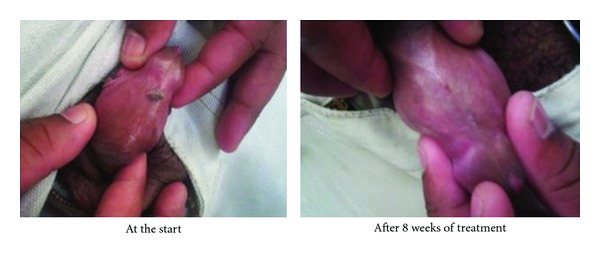
Photodocumentation. Lesions on the back of the penis.

**Figure 6:**
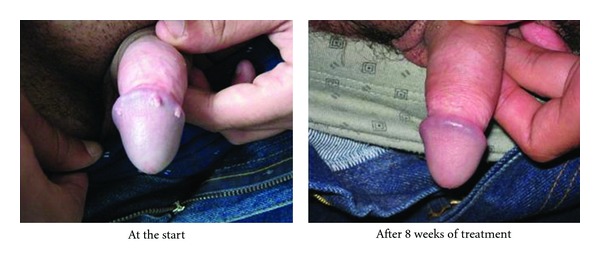
Photodocumentation. Lesions on the glans penis.

The evolution of the lesions after applying the medicine is shown in the photos (Figures 5 and 6).

## 4. Discussion

In general, response to both treatments was good, although that of Glizigen was slightly better than that of podophyllin. An analysis was carried out to see whether this difference was statistically significant for a 95% confidence interval; however the corresponding results showed it was not. International studies have produced varied efficacy responses to podophyllin that range from between 44% and 76% [1, 4, 7, 19, 24–26] and they have had good results with high rates of healing with Glizigen, although the percentage of efficacy has not been determined [27–31]. 

Given the maximum time period established to use both products, in weeks (8 for Glizigen and 6 for podophyllin), more patients whose lesions did actually heal within this period did so in the last two weeks. In the studies analysed, we did not find any specific results that were similar to those shown in our graphs, although the time limit set for the lesions to heal in the population mean for each treatment was the same.

Even though the affected area of skin was measured in cm^2^ in our clinical trial, which is different from the measuring unit of this variable used in other published studies, they do coincide in some respects. The majority of patients from most of the series analysed were reported to have less than 10 lesions (mainly small ones), which is understood as being a similarity. This still has to be proved by further research, [1, 7, 10, 19, 25, 26] but it could be due to the fact that there are plenty of qualified personnel available, the medical culture of the patients themselves, and because the lesions are very noticeable, especially for members of the sexually active population, so they go to the doctor before the affected area gets worse. In turn, an analysis was carried out to see whether the size of the affected area was associated with the response to the treatment. The results of such showed that these variables were independent too, as shown in the corresponding table.

The results obtained when using Glizigen coincide with those obtained by other authors using this medicine [19, 31, 32]. 

Out of all the findings, the penis was the most affected genital organ in men. Almost half the patients had suffered from the lesions for 1 to 4 months, which coincides with the findings of the international reports [10, 19, 25, 26, 33]. We can conclude the following.

1. Using Glizigen together with Viusid was effective in treating the external anogenital warts.
2. The variables concerning age, gender, anatomical location, and the area affected by the lesions coincide with what has been reported by other researchers.
3. There was a good clinical response to both treatments.
4. The combined Glizigen-Viusid treatment was better than the treatment with podophyllin.
5. The effectiveness and innocuousness of Glizigen combined with Viusid in the treatment of this disease are confirmed.

## Figures and Tables

**Table 1:** Distribution of patients according to their ages.

Age groups (years)	Glizigen		Podophyllin	
	No.	**%**	No.	**%**
15 to 24	29	**60.4**	30	**60**
25 to 34	10	20.8	10	20
35 to 44	3	6.3	5	10
45 to 54	2	4.2	3	6
≥55	4	8.3	2	4

Total	48	100	50	100

**Table 2:** Correlation between the cured lesion and the affected area of skin.

Area affected by the lesions	Glizigen		Podophyllin	
	Cured	Not cured	Cured	Not cured
<5 cm^2^	38	5	36	10
>5 cm^2^	4	1	2	2

Total	42	6	38	12

CI (95%)	χ^2^= 0.0319	*P* = 0.8583	χ^2^= 0.4344	*P* = 0.5098

**Table 3:** Discomfort associated with the treatment.

Symptoms	Glizigen		Podophyllin	
	No.	%	No.	**%**
Burning	3	6	15	**30**
Pain	1	2	4	8
Itching	5	10	4	8
None	39	**82**	27	**54**

Total	48	100	50	100

## References

[1] Douglas RL,  Warts JAE, Freedberg I, Eisen A, Fitzpatrick T (2001). *Dermatología en Medicina General*.

[2] Papiloma ZL http://www.facmed.unam.com.

[3] Consenso de Papiloma Virus Humano (HPV) y Herpes Simples Virus (HSV) Genital http://www.sad.org.ar.

[4] Menéndez M, Hernández MDLÁ, vela de Oro OA, Torres Chávez A (2004). Actualización de la terapéutica del papilomavirus humano. Terapia convencional. *Revista Cubana de Medicina*.

[5] Wincheids OEF, Gonzáles AL http://www.fisterra.com.br.

[7] Centers for Disease Control and Prevention (2002). Guidelines for treatment of sexually transmitted diseases. *Morbidity and Mortality Weekly Report*.

[8] Castle PE, Wacholder S, Lorincz AT (2002). A prospective study of high-grade cervical neoplasia risk among human papillomavirus-infected women. *Journal of the National Cancer Institute*.

[9] De Clercq E (1993). Therapeutic potential of HPMPC as an antiviral drug. *Reviews in Medical Virology*.

[10] Favre M, Ramoz N, Orth G (1997). Human papillomaviruses: general features. *Clinics in Dermatology*.

[11] Centers for Disease Control and Prevention http://www.cdc.gov/STD/treatment/.

[12] Van Cutsem E, Snoeck R, Van Ranst M (1995). Successful treatment of a squamous papilloma of the hypopharynx-esophagus by local injections of (S)-1-(3-hydroxy-2-phosphonylmethoxypropyl)cytosine. *Journal of Medical Virology*.

[13] Li YH, Gao XH, He CD, Guomei Z, Don ZX, Chan HD (2001). Detection of human papillomavirus and response to oral aratinoid ethylester in 2 cases of Darier disease. *Archives of Dermatology*.

[14] Bosch FX, Manos MM, Muñoz N (1995). Prevalence of human papilloma virus in cervical cancer: a worldwide perspective. *Journal of the National Cancer Institute*.

[15] Calisto D, Arcangeli F (2003). Topical cidofovir for condylomata acuminata of the genitalia in a 3-year-old child. *Journal of the American Academy of Dermatology*.

[16] Muñoz N, Bosch FX, de Sanjosé S (2003). Epidemiologic classification of human papillomavirus types associated with cervical cancer. *The New England Journal of Medicine*.

[17] Al-Gurairi FT, Al-Waiz M, Sharquie KE (2002). Oral zinc sulphate in the treatment of recalcitrant viral warts: randomized placebo-controlled clinical trial. *British Journal of Dermatology*.

[18]  Signore RJ (2001). Candida immunotherapy of warts. *Archives of Dermatology*.

[19] Mungía MA (2006). *Eficacia del ácido glicirricínico activado, con relación al nitrógeno líquido, y a la combinación de ambos, en el tratamiento del condiloma acuminado en pacientes atendidos en el Centro Nacional de Dermatología [Tesis de Especialista]*.

[20] Soto Y, Mune M, Goicolea A (1998). Aplicación de la técnica de reacción en cadena de la polimerasa para la detección de secuencias de Papillomavirus humano. *Revista Cubana de Medicina Tropical*.

[21] Sanabria GJN, Mérida AM, Medina SV, Valdés PG http://www.conganat.org/7congreso/trabajo.asp?idtrabajo=495.

[22] Sintes RÁ (2001). *Editorial Ciencias Médicas*.

[23] Colectivo de Autores MinisteriodeSaludPública (2004). *Infecciones de Transmisión Sexual, pautas para su tratamiento*.

[24] von Krogh G, Lacey CJN, Gross G, Barrasso R, Schneider A (2000). European course on HPV associated pathology: guidelines for primary care physicians for the diagnosis and management of anogenital warts. *Sexually Transmitted Infections*.

[25] Acevedo AS L (2005). *Eficacia de la Podofilina, el Ácido tricloroacético y la Criocirugía en el Tratamiento de las Verrugas Genitales Externas [Tesis de Especialista]*.

[26] Miracco C, Palummo N, Lavergne D, Nyongo A, Tosi P, de Villiers E-M (2001). Malignant melanoma: search for human papillomavirus. *Archives of Dermatology*.

[27] Luis BA (1997). *Estudio multicéntrico, prospectivo, abierto, controlado, sobre la eficacia, la seguridad clínica y la tolerabilidad local de la administración repetitiva (t.i.d.) por 5 días del ácido glicirricínico activado (Epigen) en pulverizaciones locales a pacientes cursando con herpes genital*.

[28] Bogatyreva II (2008). *Experience in Usage of EPIGEN at Treatment of Papilloma Virus Infection of the Urogenital Duct*.

[29] Martin-Crevillente O *Report on the Results of the Tests with the Product Epigen (Laboratories Cheminova International, S.A., Madrid). Evaluate the Effectiveness and the Tolerance of the Antiviral Preparation Epigen in Women with Papillomavirus Infection*.

[30] Espinoza A, Barragán L (2004). *Estudio longitudinal, prospectivo, abierto, sobre la evaluación de la efectividad, la seguridad clínica y la tolerabilidad local de la administración repetitiva (q.id.) por 10 días del ácido glicirricínico activado (Epigén) en pulverizaciones locales a pacientes cursando con infección por virus del papiloma humano (HPV) a nivel del cérvix uterino*.

[31] Zelenková H, Nejdková A, Škutilová E, Urbáni M, Švecová D, Cabalová J (2005). Preparations containing glycyrrhizic acid employed in dermatovenereologic practice. Conclusions of an international multicentre study. *Dermatologia Kliniczna*.

[32] Andrews G, Domonkos A (2004). Enfermedades víricas. *Tratado de Dermatología*.

[33] Rook A, Wilkinson DS, Ebling FJ, Champion RH, Burton J (1992). *Textbook of Dermatology*.

